# Endoscopic Papillectomy May Be a Feasible Treatment Option for Elderly Patients With Ampullary Tumors: A Multicenter Retrospective Study

**DOI:** 10.1111/den.70284

**Published:** 2026-09-10

**Authors:** Noriki Kasuga, Yusuke Kurita, Yusuke Sekino, Keiki Nagai, Yuji Fujita, Riko Hemmi, Daiki Imanaga, Manato Kaneda, Shota Matsumoto, Masato Suzuki, Shin Yagi, Kensuke Kubota, Masato Yoneda

**Affiliations:** ^1^ Department of Gastroenterology Yokohama Rosai Hospital Yokohama Japan; ^2^ Department of Gastroenterology and Hepatology Yokohama City University Hospital Yokohama Japan; ^3^ Department of Hepato‐Biliary‐Pancreatic Medicine NTT Medical Center Tokyo Tokyo Japan

**Keywords:** ampullary tumor, complication, elderly patients, endoscopic papillectomy

## Abstract

**Objectives:**

Endoscopic papillectomy (EP) is a minimally invasive treatment for ampullary tumors; however, its safety in elderly patients remains unclear. We aimed to evaluate the feasibility and clinical outcomes of EP in this population.

**Methods:**

This multicenter retrospective study included 255 patients with ampullary tumors who underwent EP between April 2008 and September 2025 at three collaborating institutions. Patients aged ≥ 75 years were defined as elderly; complication rates and clinical outcomes were compared with the non‐elderly group.

**Results:**

The elderly group had a significantly higher Charlson Comorbidity Index (1.0 [0–8] vs. 0 [0–7]; *p* = 0.028). The elderly group demonstrated significantly lower rates of overall complications (29.0% vs. 48.9%; *p* = 0.004), delayed bleeding (4.3% vs. 23.1%; *p* < 0.001), and pancreatitis (11.6% vs. 23.1%; *p* = 0.041) than the non‐elderly group. Serious complications rate (4.3% vs. 4.8%; *p* = 1.000), median hospital stay (12 [7–73] vs. 13 [4–40] days; *p* = 0.408) were comparable between groups. Multivariate analysis showed that age ≥ 75 years was independently associated with a reduced risk of delayed bleeding (*p* = 0.002; odds ratio [OR], 0.14) and pancreatitis (*p* = 0.029; OR, 0.39).

**Conclusions:**

EP in patients aged ≥ 75 years was associated with lower complication rates and may be a feasible treatment option.

## Graphical abstract

Multicenter retrospective analysis of 255 patients undergoing endoscopic papillectomy demonstrated significantly lower rates of delayed bleeding and post‐EP pancreatitis in patients aged ≥ 75 years. Age ≥ 75 75 years was independently associated with reduced risks of both adverse events, while serious complications, complete resection, and recurrence were comparable between elderly and non‐elderly patients.

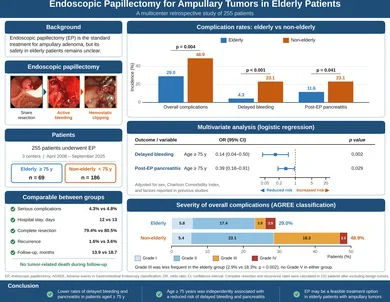

## 1 Introduction

Ampullary tumors, including adenomas and carcinomas, carry a risk of malignant transformation through the adenoma–carcinoma sequence [1]. Endoscopic papillectomy (EP), first introduced by Suzuki et al. in 1983 [2], is the standard treatment for ampullary adenomas [3, 4, 5]. For suspected carcinoma, pancreaticoduodenectomy (PD) or transduodenal ampullectomy are also used.

EP may be feasible for selected early‐stage carcinomas [6, 7], indicating expanding indications and increasing demand. EP can serve as a diagnostic tool given the suboptimal accuracy of endoscopic biopsy (25%–50% false‐negative rates [8, 9], and 38.3%–85% accuracy [10, 11, 12, 13, 14, 15]) and is sometimes performed as a “total biopsy.”

Ampullary tumors are more common in men, with a peak incidence at 61–71 years [16, 17, 18, 19]; treatment is frequently required in older adults. Age is a risk factor for mortality post‐PD [20], with mortality rates of 0%–25% and complication rates of 33%–60% [21, 22, 23, 24, 25, 26, 27]. Particularly, patients aged ≥ 75 years have a higher mortality risk post‐PD (odds ratio [OR], 5.67) [28]. Given that surgery carries higher complication rates than EP [4, 29, 30], minimally invasive approaches such as EP may be preferable in elderly patients.

However, EP carries risk, with an overall early complication rate of 21%–36% [5, 31, 32, 33]—including bleeding, pancreatitis, cholangitis, and perforation—relatively high for an endoscopic procedure. Severe and occasionally fatal pancreatitis has been reported, a concern heightened in elderly patients with a higher comorbidity burden; however, current guidelines do not clearly define EP indications in elderly patients. Notably, a recent review dedicated to EP in elderly patients also concluded that no validated algorithm for treatment selection in this population currently exists [34]. Therefore, we aimed to evaluate the incidence of complications in elderly patients undergoing EP.

## 2 Methods

### 2.1 Patients

This retrospective study included 255 patients with ampullary tumors who underwent EP between April 2008 and September 2025 at Yokohama Rosai Hospital, Yokohama City University Hospital, and NTT Medical Center, Tokyo. The study complied with the Declaration of Helsinki for biomedical research involving human participants and was approved by the relevant institutional review boards (Approval No. 2023‐60‐2).

### 2.2 EP Indication

EP was performed according to guidelines of the Japan Gastroenterological Endoscopy Society (JGES) [3]. Adenomas confirmed by preoperative biopsy were considered an absolute indication for EP. Additionally, patients with suspected ampullary adenocarcinoma without endoscopic features of ulceration or invasion were treated with EP as a relative indication, based on strong patient preference. Pre‐EP, all patients underwent endoscopic ultrasound and/or magnetic resonance cholangiopancreatography (MRCP) to confirm the absence of invasion into the duodenal muscularis, bile duct, or pancreatic duct.

### 2.3 EP Procedure

**FIGURE 1:**
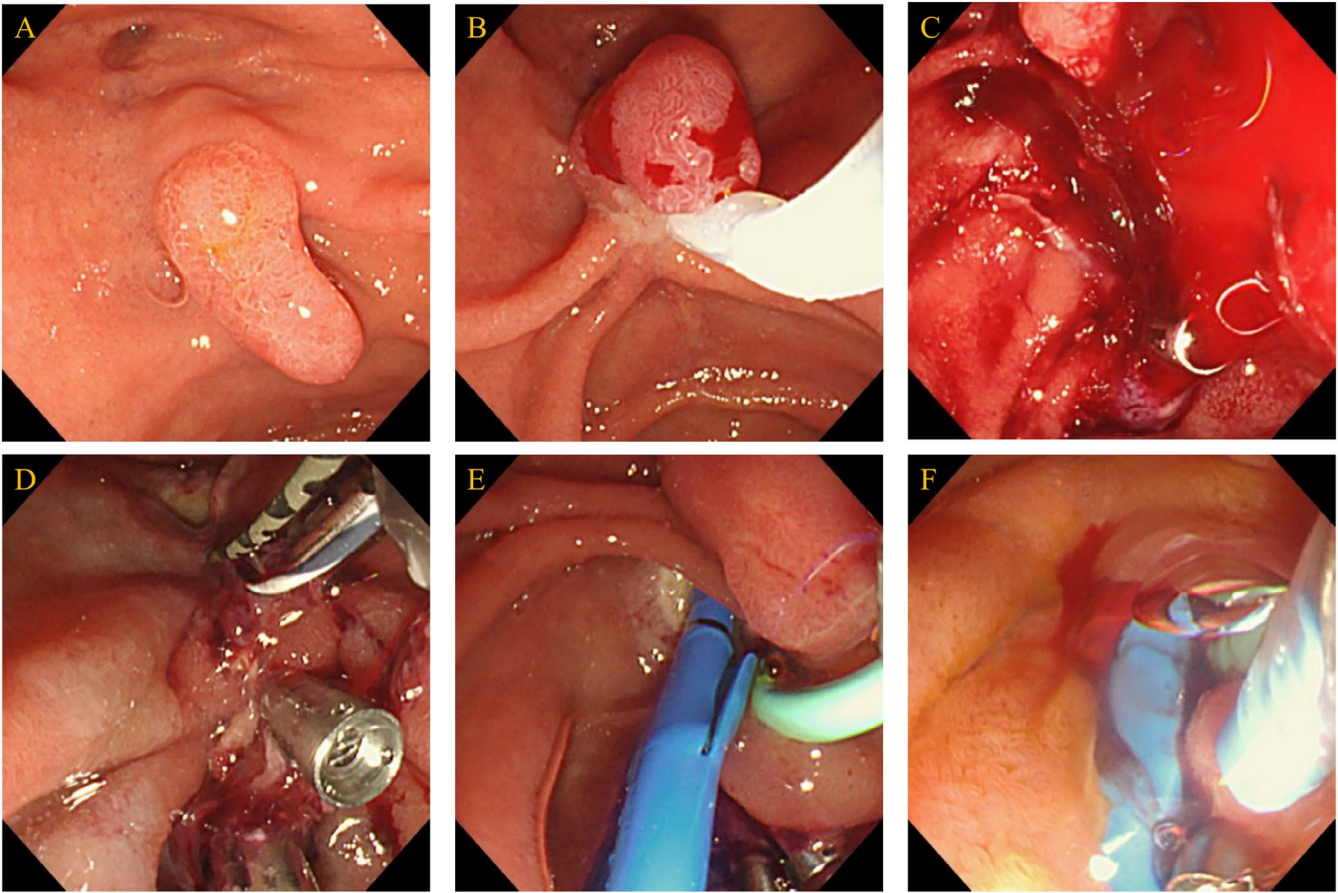
Representative case of active bleeding post‐EP. (A) En‐face view of the ampullary tumor. (B) Resection of an ampullary tumor using a snare. (C) Immediate bleeding from the post‐EP ulcer. (D) Hemostasis achieved with additional clipping. (E) Placement of biliary and pancreatic stents. (F) Application of a self‐assembling peptide hemostatic agent to achieve complete hemostasis. EP, endoscopic papillectomy.

EP was performed using a standardized endoscopic technique (Table S1). In cases of intra‐procedural bleeding, hemostasis was achieved using argon plasma coagulation (APC), clipping, or topical hemostatic agents at the endoscopist's discretion (Figure 1).

### 2.4 Definition of Complications and Outcomes

The diagnosis of delayed bleeding was defined according to the JGES guideline (2021) [3]; post‐EP pancreatitis was based on the Cotton criteria. Duodenal perforation was defined as the presence of free air in the peritoneal or retroperitoneal space. Cholangitis and cholecystitis were diagnosed according to the Tokyo Guidelines 2018 [35, 36]. Details of each complication are provided in Table S2. Post‐EP pancreatitis severity was graded according to the Cotton criteria. EP‐related complication severity was evaluated according to the Adverse events in Gastrointestinal Endoscopy (AGREE) classification [37] (Table S3). In cases of delayed bleeding, endoscopic hemostasis, including clipping, thermal coagulation, or injection therapy with hypertonic saline epinephrine, was performed as the first‐line treatment. If endoscopic hemostasis was unsuccessful, angiographic embolization or surgical intervention was considered. Complete resection was defined as histologically negative horizontal and vertical margins without evidence of lymphovascular invasion. A recurrent lesion was defined as a tumor detected during the second or subsequent endoscopic follow‐up examination, or as metastatic disease identified on other imaging modalities. A residual lesion was defined as a tumor suspected during the initial endoscopic follow‐up examination.

### 2.5 Endpoints

Patients aged ≥ 75 years were classified as elderly. The primary endpoint was the incidence of complications in the elderly and non‐elderly groups; the secondary endpoint was treatment outcomes.

### 2.6 Statistical Analysis

Statistical analysis was conducted using the chi‐squared test or Fisher's exact test, as appropriate, for categorical variables. Continuous variables were analyzed using the Student's *t*‐test or the Mann–Whitney *U* test for univariate comparisons. The Student's *t*‐test was applied when a normal distribution was assumed, whereas the Mann–Whitney *U* test was used when normality could not be confirmed. Multivariate analysis was performed using logistic regression. Variables with *p* < 0.05 in univariate analysis, the Charlson Comorbidity Index (CCI), or factors identified in previous studies as potentially relevant, were included in the multivariate model. Statistical significance was set at *p* < 0.05. All analyses were conducted using SPSS version 29 (IBM Corp., Armonk, NY, USA).

## 3 Results

### 3.1 Patient Characteristics

Characteristics of 255 patients who underwent EP are presented in Table 1. The overall median age was 67 [24–89] years; 36.9% (94/255) were women. Regarding comorbidities, the elderly group had a significantly higher rate of hypertension (62.3% vs. 30.6%; *p* < 0.001), chronic respiratory disease (7.2% vs. 1.6%; *p* = 0.036), and CCI (1.0 [0–8] vs. 0 [0–7]; *p* = 0.028) than the non‐elderly group. No significant differences were observed between groups in terms of other medical histories. Overall, 37 (14.5%) patients were receiving antithrombotic therapy, and continued aspirin use was observed in 2.0% (5/255), with no significant differences between groups. Regarding medication, proton pump inhibitor or potassium‐competitive acid blocker use was significantly more frequent in the elderly group than in the non‐elderly group (20.3% vs. 10.2%; *p* = 0.033).

**TABLE 1:** Clinical characteristics of patients who underwent endoscopic papillectomy (*n* = 255).

Variables	Overall (*n* = 255)	Elderly (*n* = 69)	Non‐elderly (*n* = 186)	*p*
Age (years), median [range]	67.0 [24–89]	78.0 [75–89]	62.0 [24–74]	< 0.001
Female sex, *n* (%)	94 (36.9)	31 (44.9)	63 (33.9)	0.104
Medical history, *n* (%)				
Hypertension	100 (39.2)	43 (62.3)	57 (30.6)	< 0.001
Diabetes	48 (18.8)	15 (21.7)	33 (17.7)	0.468
Dyslipidemia	68 (26.7)	19 (27.5)	49 (26.3)	0.848
Chronic heart disease	32 (12.5)	12 (17.4)	20 (10.8)	0.155
Cerebrovascular disease	14 (5.5)	5 (7.2)	9 (4.8)	0.536
Chronic kidney disease	10 (3.9)	4 (5.8)	6 (3.2)	0.466
Chronic respiratory disease	8 (3.1)	5 (7.2)	3 (1.6)	0.036
Cholecystectomy	9 (3.5)	5 (7.2)	4 (2.2)	0.063
Pancreas divisum	7 (2.7)	1 (1.4)	6 (3.2)	0.678
FAP	11 (4.3)	1 (1.4)	10 (5.4)	0.298
Medications, *n* (%)				
Antithrombotic agents	37 (14.5)	14 (20.3)	23 (12.4)	0.110
Anticoagulant agents	13 (5.1)	4 (5.8)	9 (4.8)	0.753
Antiplatelet agents	24 (9.4)	10 (14.5)	14 (7.5)	0.091
Continuation of aspirin	5 (2.0)	2 (2.9)	3 (1.6)	0.614
NSAID	3 (1.2)	1 (1.4)	2 (1.1)	1.000
Steroid	3 (1.2)	0 (0)	3 (1.6)	0.565
PPI or P‐CAB	33 (12.9)	14 (20.3)	19 (10.2)	0.033
CCI, median [range]	1.0 [0–8]	1.0 [0–8]	0 [0–7]	0.028

Abbreviations: CCI, Charlson Comorbidity Index; FAP, familial adenomatous polyposis; NSAID, nonsteroidal anti‐inflammatory drug; P‐CAB, potassium‐competitive acid blocker; PPI, proton pump inhibitor.

### 3.2 EP Techniques

EP techniques are summarized in Table 2. En bloc resection was achieved in 93.7% of patients (239/255); the remaining patients underwent piecemeal resection due to residual lesions. Prophylactic clipping was performed in 87.8% (224/255), and the mean overall number of clips used was 2.12 ± 1.50. APC was performed in 4.7% (12/255), thrombin was used in 11.0% (28/255), and PuraStat, a self‐assembling peptide hemostatic agent, was applied in 14.9% (38/255) for the management of immediate bleeding. The success rate of biliary stenting was 96.1% (245/255), and that of pancreatic stenting was 94.1% (240/255). The median procedure time was 32 [15–140] minutes. No significant differences in EP techniques were observed between elderly and non‐elderly groups.

**TABLE 2:** Comparison of techniques for endoscopic papillectomy (*n* = 255).

Variables	Overall	Elderly	Non‐elderly	*p*
	*n* = 255	*n* = 69	*n* = 186	
En bloc resection, *n* (%)	239 (93.7)	66 (95.7)	173 (93.0)	0.569
Snare size, *n* (%)				0.785
13 mm	148 (58.0)	41 (59.4)	107 (57.5)	
≥ 20 mm	107 (42.0)	28 (40.6)	79 (42.5)	
Prophylactic clipping, *n* (%)	224 (87.8)	59 (85.5)	165 (88.7)	0.487
Number of clips, Mean ± SD	2.12 ± 1.50	2.20 ± 1.54	2.09 ± 1.48	0.387
Additional EST, *n* (%)	14 (5.5)	6 (8.7)	8 (4.3)	0.214
APC for bleeding, *n* (%)	12 (4.7)	6 (8.7)	6 (3.2)	0.092
Hemostatic agent, *n* (%)				
Thrombin	28 (11.0)	5 (7.2)	23 (12.4)	0.245
Self‐assembling peptide	38 (14.9)	10 (14.5)	28 (15.1)	0.911
Biliary stenting, *n* (%)	245 (96.1)	65 (94.2)	180 (96.8)	0.466
Pancreatic stenting, *n* (%)	240 (94.1)	68 (98.6)	172 (92.5)	0.077
Procedure time (min), median [range]	32 [15–140]	33 [18–120]	32 [15–140]	0.531

Abbreviations: APC, argon plasma coagulation; EST, endoscopic sphincterotomy; SD, standard deviation.

### 3.3 Pathological Findings and Outcomes Post‐EP

Outcomes of final diagnosis and follow‐up post‐EP are presented in Table 3. The most frequent tumor type was adenoma (77.6%, 198/255), followed by adenocarcinoma (12.9%, 33/255), benign tumors (9.0%, 23/255), and neuroendocrine tumors (0.4%, 1/255). Adenocarcinoma was more common in the elderly group than in the non‐elderly group, although this difference was not statistically significant (18.8% vs. 10.8%, *p* = 0.087). The median tumor size was 10.0 [3.0–26.0] mm. Among 232 patients with adenoma, adenocarcinoma, or neuroendocrine tumors (i.e., excluding benign tumors), the complete resection rate was 80.2% (186/232), the residual rate was 5.2% (12/232), and the recurrence rate was 3.0% (7/232). The median follow‐up period was 18.2 [0.2–138.9] months; the time to recurrence was 16.1 [7.1–41.5] months. There were no tumor‐related deaths; no significant differences were observed in pathological findings or follow‐up outcomes between elderly and non‐elderly patients undergoing EP.

**TABLE 3:** Pathological findings and outcomes for endoscopic papillectomy.

Pathological findings (*n* = 255)				
Variables	Overall	Elderly	Non‐elderly	*p*
	*n* = 255	*n* = 69	*n* = 186	
Final diagnosis, *n* (%)				
Adenoma	198 (77.6)	50 (72.5)	148 (79.6)	0.226
Adenocarcinoma	33 (12.9)	13 (18.8)	20 (10.8)	0.087
Benign^a^	23 (9.0)	6 (8.7)	17 (9.1)	0.912
Neuroendocrine tumor	1 (0.4)	0 (0)	1 (0.5)	1.000
Tumor size (mm), median [range]	10.0 [3.0–26.0]	10.0 [3.0–25.0]	10.0 [3.0–26.0]	0.230
Outcomes (*n* = 232)				
	***n* = 232**	***n* = 63**	***n* = 169**	
Complete resection, *n* (%)	186 (80.2)	50 (79.4)	136 (80.5)	0.851
Additional treatment, *n* (%)				
Surgical resection	8 (3.4)	3 (4.8)	5 (3.0)	0.451
Endoscopic treatment	14 (6.0)	3 (4.8)	11 (6.5)	0.619
Residual, *n* (%)	12 (5.2)	2 (3.2)	10 (5.9)	0.521
Recurrence, *n* (%)	7 (3.0)	1 (1.6)	6 (3.6)	0.678
Follow‐up period (months), median [range]	18.2 [0.2–138.9]	13.9 [0.2–93.2]	18.7 [0.4–138.9]	0.250
Follow‐up mortality, *n* (%)	5 (2.2)	2 (3.2)	3 (1.8)	0.615
Tumor‐related death	0	0	0	N.S.
Non tumor‐related death	5 (2.2)	2 (3.2)	3 (1.8)	0.615
Time to recurrence (months), median [range]	16.1 [7.1–41.5]	23.5^b^	12.2 [7.1–41.5]	0.750

^a^ “Benign” refers to hyperplasia, papillitis, inflammatory granuloma, and gangliocytic paraganglioma.

^b^ Time to recurrence in the elderly group is based on a single case (*n* = 1); range is not applicable.

### 3.4 Comparison of Early Complications Following EP

As shown in Table 4, the elderly group had a significantly lower rate of overall complications (29.0% vs. 48.9%; *p* = 0.004), delayed bleeding (4.3% vs. 23.1%; *p* < 0.001) and pancreatitis (11.6% vs. 23.1%; *p* = 0.041) than the non‐elderly group following EP. In the severity assessment using the AGREE classification, Grade III was significantly less frequent in the elderly group (2.9% vs. 18.3%; *p* = 0.002). Regarding the severity of delayed bleeding, Grade III was significantly more frequent in the non‐elderly group (1.4% vs. 18.3%; *p* < 0.001). Angioembolization was required in 1.2% patients (3/255) for management of delayed bleeding. The incidence of Grade II post‐EP pancreatitis was significantly lower in the elderly group (10.1% vs. 21.0%; *p* = 0.046); severe pancreatitis based on the Cotton criteria did not differ significantly between the two groups (2.9% vs. 3.2%; *p* = 1.000). Duodenal perforation occurred more frequently in the elderly group than in the non‐elderly group (4.3% vs. 0.5%; *p* = 0.062), although this difference was not statistically significant. In one case requiring emergency surgery in the elderly group, perforation of the duodenal mucosa on the contralateral side of the papilla occurred during advancement of the clipping device, rather than during tumor resection. Although this event was not fatal, it resulted in prolonged hospitalization. There were no significant differences in biliary tract infections (2.9% vs. 2.7%; *p* = 1.000) or length of hospital stay (12 [7–73] vs. 13 [4–40] days; *p* = 0.408) between groups; no EP‐related deaths (i.e., AGREE Grade V) were observed. Defining “serious complications” as bleeding requiring embolization, severe pancreatitis based on the Cotton criteria, and duodenal perforation requiring surgery revealed no significant difference between the two groups (4.3% vs. 4.8%; *p* = 1.000).

**TABLE 4:** Comparison of early complications and clinical outcomes for endoscopic papillectomy (*n* = 255).

Variables	Overall	Elderly	Non‐elderly	*p*
	*n* = 255	*n* = 69	*n* = 186	
Overall complication^a^, *n* (%)	111 (43.5)	20 (29.0)	91 (48.9)	0.004
AGREE Grade I	14 (5.5)	4 (5.8)	10 (5.4)	1.000
AGREE Grade II	55 (21.6)	12 (17.4)	43 (23.1)	0.323
AGREE Grade III	36 (14.1)	2 (2.9)	34 (18.3)	0.002
AGREE Grade IV	6 (2.4)	2 (2.9)	4 (2.2)	0.663
AGREE Grade V	0 (0)	0 (0)	0 (0)	N.S.
Immediate bleeding, *n* (%)	34 (13.3)	9 (13.0)	25 (13.4)	0.934
Delayed bleeding, *n* (%)	46 (18.0)	3 (4.3)	43 (23.1)	< 0.001
AGREE Grade I	3 (1.2)	0 (0)	3 (1.6)	0.565
AGREE Grade II	4 (1.6)	1 (1.4)	3 (1.6)	1.000
AGREE Grade III	35 (13.7)	1 (1.4)	34 (18.3)	< 0.001
AGREE Grade IV	4 (1.6)	1 (1.4)	3 (1.6)	1.000
AGREE Grade V	0 (0)	0 (0)	0 (0)	N.S.
Angioembolization, *n* (%)	3 (1.2)	0 (0)	3 (1.6)	0.565
Pancreatitis, *n* (%)	51 (20.0)	8 (11.6)	43 (23.1)	0.041
AGREE Grade I	3 (1.2)	0 (0)	3 (1.6)	0.565
AGREE Grade II	46 (18.0)	7 (10.1)	39 (21.0)	0.046
AGREE Grade III	0 (0)	0 (0)	0 (0)	N.S.
AGREE Grade IV	2 (0.8)	1 (1.4)	1 (0.5)	0.469
AGREE Grade V	0 (0)	0 (0)	0 (0)	N.S.
Severe pancreatitis^b^	8 (3.1)	2 (2.9)	6 (3.2)	1.000
Duodenal perforation, *n* (%)	4 (1.6)	3 (4.3)	1 (0.5)	0.062
AGREE Grade I	0 (0)	0 (0)	0 (0)	N.S.
AGREE Grade II	3 (1.2)	2 (2.9)	1 (0.5)	0.179
AGREE Grade III	0 (0)	0 (0)	0 (0)	N.S.
AGREE Grade IV	1 (0.4)	1 (1.4)	0 (0)	0.271
AGREE Grade V	0 (0)	0 (0)	0 (0)	N.S.
Emergency surgery, *n* (%)	1 (0.4)	1 (1.4)	0 (0)	0.271
Biliary tract infections, *n* (%)	7 (2.7)	2 (2.9)	5 (2.7)	1.000
AGREE Grade I	1 (0.4)	0 (0)	1 (0.5)	1.000
AGREE Grade II	3 (1.2)	1 (1.4)	2 (1.1)	1.000
AGREE Grade III	3 (1.2)	1 (1.4)	2 (1.1)	1.000
AGREE Grade IV	0 (0)	0 (0)	0 (0)	N.S.
AGREE Grade V	0 (0)	0 (0)	0 (0)	N.S.
Serious complications^c^, *n* (%)	12 (4.7)	3 (4.3)	9 (4.8)	1.000
Hospital stay (days), median [range]	12 [4–73]	12 [7–73]	13 [4–40]	0.408

Abbreviations: AGREE, adverse events in gastrointestinal endoscopy; N.S., not significant.

^a^ Overall complications included immediate bleeding, delayed bleeding, pancreatitis, cholangitis, cholecystitis, and perforation.

^b^ Cotton criteria.

^c^ Serious complications included bleeding requiring embolization, severe pancreatitis (Cotton criteria), and duodenal perforation requiring surgery.

### 3.5 Multivariate Analysis of Factors Influencing Delayed Bleeding and Pancreatitis

Univariate and multivariate analyses were performed to identify factors associated with delayed bleeding and pancreatitis based on patient characteristics and EP techniques (Tables 5 and 6). Regarding delayed bleeding, age ≥ 75 years (*p* < 0.001), female sex (*p* = 0.019), and anticoagulant use (*p* = 0.003) were identified in univariate analysis and were subsequently included in the multivariate model based on a previous report [38], along with CCI. Age ≥ 75 years was independently associated with a reduced risk of delayed bleeding (*p* = 0.002; OR, 0.14; 95% confidence interval [CI], 0.04–0.50), whereas anticoagulant use was associated with an increased risk (*p* = 0.006; OR, 7.24; 95% CI, 1.76–29.83). Regarding pancreatitis, age ≥ 75 years (*p* = 0.041) was identified in univariate analysis; “female sex” and “pancreatic stenting” were added to the multivariate model based on a previous report [39], along with CCI. In multivariate analysis, age ≥ 75 years was independently associated with a reduced risk of pancreatitis (*p* = 0.029; OR, 0.39; 95% CI, 0.18–0.91).

**TABLE 5:** Factors influencing delayed bleeding following endoscopic papillectomy (*n* = 255).

Factors	Delayed bleeding	Univariate analysis			Multivariate analysis		
		OR	95% CI	*p*	OR	95% CI	*p*
Age ≥ 75 years	4.3% (3/69)	0.15	0.05–0.51	< 0.001	0.14	0.04–0.50	0.002
Female sex	10.6% (10/94)	0.41	0.19–0.88	0.019	0.49	0.23–1.07	0.074
CCI ≥ 3	31.8% (7/22)	2.32	0.89–6.07	0.087	1.06	0.32–3.55	0.920
Pancreas divisum	14.3% (1/7)	0.75	0.09–6.40	1.000	−	−	−
Anticoagulant agents	53.8% (7/13)	6.07	1.94–19.05	0.003	7.24	1.76–29.83	0.006
Antiplatelet agents	8.3% (2/24)	0.39	0.09–1.71	0.269	−	−	−
Continuation of aspirin	20.0% (1/5)	1.14	0.12–10.43	1.000	−	−	−
NSAID	0% (0/3)	−	−	1.000	−	−	−
Steroid	66.7% (2/3)	9.46	0.84–106.58	0.085	−	−	−
PPI or P‐CAB	15.2% (5/33)	0.79	0.29–2.17	0.644	−	−	−
En bloc resection	18.8% (45/239)	3.48	0.45–27.03	0.318	−	−	−
Snare size ≥ 20 mm	17.8% (19/107)	0.97	0.51–1.85	0.921	−	−	−
Prophylactic clipping	17.9% (40/224)	0.91	0.35–2.35	0.839	−	−	−
Additional EST	7.1% (1/14)	0.34	0.04–2.63	0.476	−	−	−
APC for bleeding	25.0% (3/12)	1.55	0.40–5.97	0.458	−	−	−
Thrombin	25.0% (7/28)	1.61	0.64–4.04	0.310	−	−	−
Self‐assembling peptide	18.4% (7/38)	1.03	0.42–2.51	0.947	−	−	−
Biliary stenting	18.4% (45/245)	2.03	0.25–16.39	0.695	−	−	−
Pancreatic stenting	18.3% (44/240)	1.46	0.32–6.70	1.000	−	−	−
Procedure time ≥ 30 min	18.2% (27/148)	1.03	0.54–1.98	0.921	−	−	−

Abbreviations: −, not analyzed; APC, Argon plasma coagulation; CCI, Charlson Comorbidity Index; CI, confidence interval; EST, endoscopic sphincterotomy; FAP, familial adenomatous polyposis; NSAID, nonsteroidal anti‐inflammatory drug; OR, odds ratio; P‐CAB, potassium‐competitive acid blocker; PPI, proton pump inhibitors.

**TABLE 6:** Factors influencing pancreatitis following endoscopic papillectomy (*n* = 255).

Factors	Pancreatitis	Univariate analysis			Multivariate analysis		
		OR	95% CI	*p*	OR	95% CI	*p*
Age ≥ 75 years	11.6% (8/69)	0.44	0.19–0.98	0.041	0.39	0.18–0.91	0.029
Female sex	23.4% (22/94)	1.39	0.75–2.60	0.299	1.59	0.84–3.04	0.153
CCI ≥ 3	27.3% (6/22)	1.57	0.58–4.23	0.403	1.58	0.58–4.35	0.375
Pancreas divisum	0% (0/7)	−	−	0.351	−	−	−
Anticoagulant agents	30.8% (4/13)	1.84	0.54–6.25	0.300	−	−	−
Antiplatelet agents	8.3% (2/24)	0.34	0.08–1.49	0.182	−	−	−
Continuation of aspirin	20.0% (1/5)	1.00	0.11–9.14	1.000	−	−	−
NSAID	0% (0/3)	−	−	1.000	−	−	−
Steroid	33.3% (1/3)	2.02	0.18–22.72	0.490	−	−	−
PPI or P‐CAB	15.2% (5/33)	0.68	0.25–1.87	0.456	−	−	−
En bloc resection	20.9% (50/239)	3.97	0.51–30.77	0.207	−	−	−
Snare size ≥ 20 mm	24.3% (26/107)	1.58	0.85–2.93	0.144	−	−	−
Prophylactic clipping	19.6% (44/224)	0.84	0.34–2.07	0.702	−	−	−
Additional EST	21.4% (3/14)	1.09	0.29–4.09	1.000	−	−	−
APC for bleeding	16.7% (2/12)	0.79	0.17–3.73	1.000	−	−	−
Thrombin	21.4% (6/28)	1.10	0.42–2.88	0.841	−	−	−
Self‐assembling peptide	13.2% (5/38)	0.56	0.21–1.52	0.253	−	−	−
Biliary stenting	20.4% (50/245)	2.31	0.29–18.64	0.692	−	−	−
Pancreatic stenting	20.4% (49/240)	1.67	0.36–7.64	0.742	2.22	0.47–10.39	0.311
Procedure time ≥ 30 min	20.3% (30/148)	1.04	0.56–1.94	0.899	−	−	−

Abbreviations: −, not analyzed; APC, argon plasma coagulation; CCI, Charlson Comorbidity Index; CI, confidence interval; EST, endoscopic sphincterotomy; FAP, familial adenomatous polyposis; NSAID, nonsteroidal anti‐inflammatory drug; OR, odds ratio; P‐CAB, potassium‐competitive acid blocker; PPI, proton pump inhibitors.

### 3.6 Sub‐Analysis of Clinical Characteristics of Delayed Bleeding

Table 7 presents the sub‐analysis of 46 cases that developed delayed bleeding. The breakdown of bleeding severity (*p* = 0.157), need for blood transfusions (33.3% vs. 16.3%; *p* = 0.444), median timing of onset (2 [2–5] vs. 2 [0–6] days; *p* = 0.345), and median number of endoscopic hemostasis procedures (1 [0–2] vs. 1 [0–3]; *p* = 0.867) did not differ significantly between the two groups. Meanwhile, the median hemoglobin drop was significantly smaller in the elderly group than in the non‐elderly group (2.2 [2.1–2.2] vs. 3.6 [1.5–9.5] g/dL; *p* = 0.003).

**TABLE 7:** Clinical characteristics of delayed bleeding after endoscopic papillectomy (*n* = 46).

Variables	Overall	Elderly	Non‐elderly	*p*
	*n* = 46	*n* = 3	*n* = 43	
Severity, *n* (%)				0.157
AGREE Grade I	3 (6.5)	0 (0)	3 (7.0)	
AGREE Grade II	4 (8.7)	1 (33.3)	3 (7.0)	
AGREE Grade III	35 (76.1)	1 (33.3)	34 (79.1)	
AGREE Grade IV	4 (8.7)	1 (33.3)	3 (7.0)	
AGREE Grade V	0 (0)	0 (0)	0 (0)	
Need for blood transfusions, *n* (%)	8 (17.4)	1 (33.3)	7 (16.3)	0.444
Timing of onset (postoperative days), median [range]	2 [0–6]	2 [2–5]	2 [0–6]	0.345
Number of endoscopic hemostasis procedures, median [range]	1 [0–3]	1 [0–2]	1 [0–3]	0.867
Hemoglobin drop (g/dL), median [range]	3.5 [1.5–9.5]	2.2 [2.1–2.2]	3.6 [1.5–9.5]	0.003

## 4 Discussion

To our knowledge, few studies have evaluated the feasibility of EP in elderly patients, including a single‐center study with a small sample size [40] and reports highlighting potential risks in elderly patients with comorbidities [41, 42]. A novel finding of this multicenter study is that incidence rates of both delayed bleeding and pancreatitis were significantly lower in patients aged ≥ 75 years than in non‐elderly patients and that age ≥ 75 years was independently associated with a reduced risk of both complications. Notably, delayed bleeding after EP has mainly been addressed through procedural preventive strategies, such as prophylactic clip closure of the post‐resection ulcer and optimization of electrosurgical unit settings or snare wire diameter [43], whereas patient‐related factors have received little attention; the present finding that elderly patients were associated with a reduced incidence of delayed bleeding adds this dimension to the issue. Furthermore, no significant difference in the hospital length of stay or serious complications was observed between groups. These observations suggest that EP is a feasible option even in elderly patients, despite their higher comorbidity burden.

In reports on endoscopic submucosal dissection (ESD), advanced age has not been reported as a risk factor for post‐ESD bleeding [44, 45]. However, in the present study, the reduced risk of delayed bleeding in elderly patients undergoing EP suggests a unique age‐related association specific to this procedure. One possible explanation is that age‐related changes in pancreatic exocrine function may contribute to reduced bleeding risk [46, 47]. Specifically, reduced exposure of post‐EP ulcers to digestive enzymes may play a role in lowering bleeding rates in elderly patients. Fukuhara et al. [48] reported that external drainage of the bile and pancreatic ducts after duodenal ESD reduced the risk of ulcer bleeding and perforation, supporting the potential importance of pancreatic and biliary secretions in post‐procedural bleeding. Another hypothesis is that blood flow around the papilla of Vater decreases with aging. In the present study, elderly patients had a higher prevalence of hypertension, which may reflect underlying atherosclerotic changes. Given reports suggesting that atherosclerosis can reduce pancreatic blood flow [49], age‐related vascular changes may indirectly influence perfusion around the papilla of Vater. Nevertheless, these mechanisms remain unconfirmed at the pathological and basic‐science levels, and whether this reduced bleeding risk in elderly patients is a reproducible phenomenon has yet to be established; further investigation, including basic and mechanistic studies, is warranted.

Age‐related changes in pancreatic exocrine function have similarly been implicated in the reportedly lower risk of post‐ERCP pancreatitis in elderly patients [50, 51]. These findings may also be relevant in the context of EP. However, other studies have reported no association between age and post‐ERCP pancreatitis [52]; pancreatic atrophy has not been specific to aging, because it has also been associated with atherosclerosis and genetic factors [53]. Therefore, the clinical significance of pancreatic atrophy remains unclear, and whether elderly patients have a reduced risk of post‐EP pancreatitis remains controversial.

Nonetheless, caution is required when performing EP in elderly patients. Although the incidence was low (≤ 5%), severe complications such as pancreatitis and duodenal perforation were observed. Because advanced age is a prognostic factor for acute‐pancreatitis severity and is an independent risk factor for mortality and organ failure, as established by the Japanese Ministry of Health, Labour, and Welfare (2008) [54, 55], careful management is necessary in elderly patients who develop pancreatitis. Furthermore, advanced age has been recognized as a risk factor for colonoscopic perforation [56, 57]. Age‐related alterations in collagen structure may reduce the mechanical strength of the colonic wall [58], and similar changes may occur in the duodenal wall. Therefore, EP should be performed with caution in elderly patients to minimize the risk of perforation. Even rare severe adverse events should not be underestimated.

Our findings also have implications for treatment selection. Pathological findings and follow‐up outcomes did not differ between elderly and non‐elderly patients undergoing EP in this study. Although surgical resection enables complete tumor removal without residual adenomatous tissue [59, 60], it is associated with substantial morbidity, including anastomotic leakage (up to 9%) and fistula formation (up to 14%), with reported postoperative mortality of 1%–9% [61, 62, 63]. By contrast, EP has been shown to be less invasive than surgery using established procedural scoring systems [30, 64], and recurrence post‐EP has been reported to be manageable with additional endoscopic treatment [30, 65]. Moreover, recent reports indicate that even adenocarcinomas up to T1a(M) may be managed with EP [6, 7, 65]. Taken together, EP may be a reasonable treatment option for endoscopically manageable ampullary tumors (e.g., adenomas or early‐stage carcinomas), particularly in elderly patients at high surgical risk.

However, the question of whether all elderly patients should undergo EP for ampullary tumors remains controversial and requires careful consideration. Takada et al. [41] reported that, based on the age‐adjusted CCI (ACCI) with a cutoff score of 5, patients with higher ACCI scores were less likely to benefit from EP, suggesting that observation may be a reasonable management option. It has also been argued that the outcomes of EP should be evaluated not only by short‐term results but also by long‐term prognosis, and that patient age and comorbidity should therefore be incorporated into the decision to intervene [66]. Additionally, the natural history of ampullary adenomas indicates that only 1.4% progress to cancer over an 8‐year follow‐up period [67]. Given the extremely slow progression of these lesions, their clinical significance may diminish with increasing age. The most appropriate candidates for EP are those with symptomatic disease, such as cholangitis, or those with suspected early‐stage carcinoma. In asymptomatic elderly patients with ampullary adenomas and multiple comorbidities, a non‐interventional approach may also be appropriate. A recent review has further proposed a practical framework in which oncologic risk and geriatric vulnerability are assessed separately, with structured surveillance considered as an option for asymptomatic lesions in patients with high geriatric vulnerability [34].

This study has some limitations. First, this was a retrospective study with a limited sample size. EP procedures varied among institutions with respect to the use of hemostasis methods, protocol for sedation, and management of complications. In addition, the possibility of a Type II error in the incidence of duodenal perforation in the elderly group cannot be ruled out. Second, computed tomography was not performed in all cases; therefore, subclinical pancreatitis may have been underdiagnosed based on the revised Atlanta classification. Third, selection bias cannot be excluded because elderly patients may have undergone more stringent selection and more cautious procedural management by endoscopists. Elderly patients selected for EP may represent a more carefully chosen subgroup with anatomically favorable lesions and lower procedural difficulty, which may partly explain the lower observed complication rates. Unknown confounding factors may also be at play; future studies should include prospective, ideally multicenter designs to validate these findings for EP in elderly patients.

In conclusion, EP in patients aged ≥ 75 years was associated with significantly lower rates of delayed bleeding and pancreatitis. Although the incidence of serious complications was comparable to that in non‐elderly patients, no mortality related to post‐EP complications was observed, and elderly patients did not adversely affect pathological or follow‐up outcomes. These findings indicate that EP may be considered a treatment option for ampullary tumors in elderly patients.

## Author Contributions

**Noriki Kasuga:** conceptualization, manuscript writing, investigation (endoscopic procedures). **Yusuke Kurita:** conceptualization, methodology, investigation (endoscopic procedures), writing – review and editing. **Yusuke Sekino, Keiki Nagai, Yuji Fujita:** investigation (endoscopic procedures), data curation, formal analysis, supervision. **Riko Hemmi, Daiki Imanaga, Manato Kaneda, Shota Matsumoto, Masato Suzuki, Shin Yagi:** investigation (endoscopic procedures), data curation. **Kensuke Kubota, Masato Yoneda:** supervision. All authors have approved the final version of the manuscript.

## Funding

The authors have nothing to report.

## Ethics Statement

This study complied with the Declaration of Helsinki and was approved by the Institutional Review Board of the participating institutions (Approval No. 2023‐60‐2).

## Consent

The requirement for written informed consent was waived because only medical information was used without invasion of participants' privacy in this multicenter retrospective observational study. Instead, all patients received an opt‐out form for informed consent, and those who did not consent were excluded.

## Conflicts of Interest

Masato Yoneda received speaker fees from Kowa related to metabolic dysfunction‐associated steatotic liver disease (MASLD), and research funding from Gilead Sciences Inc. related to MASLD. The other authors declare no conflicts of interest.

## Supporting information


**Table S1:** Details of EP procedure.
**Table S2:** Definitions of EP‐related complications.
**Table S3:** AGREE classification for grading the severity of adverse events.

## Data Availability

The data that support the findings of this study are available from the corresponding author upon reasonable request.

## References

[1] M. Stolte and C. Pscherer , “Adenoma‐Carcinoma Sequence in the Papilla of Vater,” Scandinavian Journal of Gastroenterology 31, no. 4 (1996): 376–382, 10.3109/00365529609006414. 8726307

[2] K. Suzuki , U. Kantou , and Y. Murakami , “Two Cases With Ampullary Cancer Who Underwent Endoscopic Excision,” Progress of Digestive Endoscopy 23 (1983): 236–239.

[3] T. Itoi , S. Ryozawa , A. Katanuma , et al., “Clinical Practice Guidelines for Endoscopic Papillectomy,” Digestive Endoscopy 34, no. 3 (2022): 394–411, 10.1111/den.14233. 35000226

[4] ASGE Standards of Practice Committee , K. V. Chathadi , M. A. Khashab , et al., “The Role of Endoscopy in Ampullary and Duodenal Adenomas,” Gastrointestinal Endoscopy 82, no. 5 (2015): 773–781, 10.1016/j.gie.2015.06.027. 26260385

[5] G. Vanbiervliet , M. Strijker , M. Arvanitakis , et al., “Endoscopic Management of Ampullary Tumors: European Society of Gastrointestinal Endoscopy (ESGE) Guideline,” Endoscopy 53, no. 4 (2021): 429–448, 10.1055/a-1397-3198. 33728632

[6] K. Yamamoto , T. Itoi , A. Sofuni , et al., “Expanding the Indication of Endoscopic Papillectomy for T1a Ampullary Carcinoma,” Digestive Endoscopy 31, no. 2 (2019): 188–196, 10.1111/den.13265. 30161275

[7] K. Suzuki , Y. Kurita , K. Kubota , et al., “Endoscopic Papillectomy Could be Rewarding to Patients With Early Stage Duodenal Ampullary Carcinoma?,” Journal of Hepato‐Biliary‐Pancreatic Sciences 31, no. 3 (2024): 203–212, 10.1002/jhbp.1398. 38014632

[8] J. Askew and S. Connor , “Review of the Investigation and Surgical Management of Resectable Ampullary Adenocarcinoma,” HPB 15, no. 11 (2013): 829–838, 10.1111/hpb.12038. 23458317 PMC4503279

[9] F. Liu , J. L. Cheng , J. Cui , et al., “Surgical Method Choice and Coincidence Rate of Pathological Diagnoses in Transduodenal Ampullectomy: A Retrospective Case Series Study and Review of the Literature,” World Journal of Clinical Cases 7, no. 6 (2019): 717–726, 10.12998/wjcc.v7.i6.717. 30968036 PMC6448071

[10] K. Yamaguchi , M. Enjoji , and K. Kitamura , “Endoscopic Biopsy Has Limited Accuracy in Diagnosis of Ampullary Tumors,” Gastrointestinal Endoscopy 36, no. 6 (1990): 588–592, 10.1016/s0016-5107(90)71170-4. 2279648

[11] N. A. Kimchi , V. Mindrul , E. Broide , and E. Scapa , “The Contribution of Endoscopy and Biopsy to the Diagnosis of Periampullary Tumors,” Endoscopy 30, no. 6 (1998): 538–543, 10.1055/s-2007-1001340. 9746162

[12] C. Rodríguez , F. Borda , I. Elizalde , F. J. Jiménez Pérez , and D. Carral , “How Accurate Is Preoperative Diagnosis by Endoscopic Biopsies in Ampullary Tumours?,” Revista Española de Enfermedades Digestivas 94, no. 10 (2002): 585–592. 12647408

[13] G. Elek , S. Győri , B. Tóth , and A. Pap , “Histological Evaluation of Preoperative Biopsies From Ampulla Vateri,” Pathology Oncology Research 9, no. 1 (2003): 32–41, 10.1007/BF03033712. 12704445

[14] S. R. Grobmyer , C. N. Stasik , P. Draganov , et al., “Contemporary Results With Ampullectomy for 29 ‘Benign’ Neoplasms of the Ampulla,” Journal of the American College of Surgeons 206, no. 3 (2008): 466–471, 10.1016/j.jamcollsurg.2007.09.005. 18308217

[15] W. Laleman , A. Verreth , B. Topal , et al., “Endoscopic Resection of Ampullary Lesions: A Single‐Center 8‐Year Retrospective Cohort Study of 91 Patients With Long‐Term Follow‐Up,” Surgical Endoscopy 27, no. 10 (2013): 3865–3876, 10.1007/s00464-013-2996-2. 23708714

[16] C. L. Cheng , S. Sherman , E. L. Fogel , et al., “Endoscopic Snare Papillectomy for Tumors of the Duodenal Papillae,” Gastrointestinal Endoscopy 60, no. 5 (2004): 757–764, 10.1016/s0016-5107(04)02029-2. 15557951

[17] S. Bohnacker , U. Seitz , D. Nguyen , et al., “Endoscopic Resection of Benign Tumors of the Duodenal Papilla Without and With Intraductal Growth,” Gastrointestinal Endoscopy 62, no. 4 (2005): 551–560, 10.1016/j.gie.2005.04.053. 16185970

[18] D. Ramai , A. Ofosu , J. Singh , F. John , M. Reddy , and D. G. Adler , “Demographics, Tumor Characteristics, Treatment, and Clinical Outcomes of Patients With Ampullary Cancer: A Surveillance, Epidemiology, and End Results (SEER) Cohort Study,” Minerva Gastroenterologica e Dietologica 65, no. 2 (2019): 85–90, 10.23736/S1121-421X.18.02543-6. 30488680

[19] S. J. Choi , H. S. Lee , J. Kim , et al., “Clinical Outcomes of Endoscopic Papillectomy of Ampullary Adenoma: A Multi‐Center Study,” World Journal of Gastroenterology 28, no. 17 (2022): 1845–1859, 10.3748/wjg.v28.i17.1845. 35633905 PMC9099193

[20] W. Kimura , H. Miyata , M. Gotoh , et al., “A Pancreaticoduodenectomy Risk Model Derived From 8575 Cases From a National Single‐Race Population (Japanese) Using a Web‐Based Data Entry System: The 30‐Day and In‐Hospital Mortality Rates for Pancreaticoduodenectomy,” Annals of Surgery 259, no. 4 (2014): 773–780, 10.1097/SLA.0000000000000263. 24253151

[21] O. F. Bathe , D. Levi , H. Caldera , et al., “Radical Resection of Periampullary Tumors in the Elderly: Evaluation of Long‐Term Results,” World Journal of Surgery 24, no. 3 (2000): 353–358, 10.1007/s002689910056. 10658072

[22] R. Scurtu , P. Bachellier , E. Oussoultzoglou , E. Rosso , R. Maroni , and D. Jaeck , “Outcome After Pancreaticoduodenectomy for Cancer in Elderly Patients,” Journal of Gastrointestinal Surgery 10, no. 6 (2006): 813–822, 10.1016/j.gassur.2005.12.010. 16769537

[23] R. Ballarin , M. Spaggiari , F. Di Benedetto , et al., ““Per le Neoplasie Infiltranti Il Duodeno Esiste Un Limite di Età Per Una Chirurgia Radicale?” [Is There an Age Limit for Radical Surgery in Case of Tumors Infiltrating the Duodenum?],” Minerva Chirurgica 65, no. 1 (2010): 1–9. 20212411

[24] Y. Ito , T. Kenmochi , T. Irino , et al., “The Impact of Surgical Outcome After Pancreaticoduodenectomy in Elderly Patients,” World Journal of Surgical Oncology 9 (2011): 102, 10.1186/1477-7819-9-102. 21906398 PMC3182908

[25] A. S. Barbas , R. S. Turley , E. P. Ceppa , et al., “Comparison of Outcomes and the Use of Multimodality Therapy in Young and Elderly People Undergoing Surgical Resection of Pancreatic Cancer,” Journal of the American Geriatrics Society 60, no. 2 (2012): 344–350, 10.1111/j.1532-5415.2011.03785.x. 22211710

[26] S. Yamada , M. Shimada , T. Utsunomiya , et al., “Surgical Results of Pancreatoduodenectomy in Elderly Patients,” Surgery Today 42, no. 9 (2012): 857–862, 10.1007/s00595-012-0169-x. 22447454

[27] Y. I. Yamashita , K. Shirabe , E. Tsujita , et al., “Surgical Outcomes of Pancreaticoduodenectomy for Periampullary Tumors in Elderly Patients,” Langenbeck's Archives of Surgery 398, no. 4 (2013): 539–545, 10.1007/s00423-013-1061-x. 23412595

[28] P. Sukharamwala , J. Thoens , M. Szuchmacher , J. Smith , and P. DeVito , “Advanced Age Is a Risk Factor for Post‐Operative Complications and Mortality After a Pancreaticoduodenectomy: A Meta‐Analysis and Systematic Review,” HPB: The Official Journal of the International Hepato Pancreato Biliary Association 14, no. 10 (2012): 649–657, 10.1111/j.1477-2574.2012.00506.x. 22954000 PMC3461370

[29] E. P. Ceppa , R. A. Burbridge , K. L. Rialon , et al., “Endoscopic Versus Surgical Ampullectomy: An Algorithm to Treat Disease of the Ampulla of Vater,” Annals of Surgery 257, no. 2 (2013): 315–322, 10.1097/SLA.0b013e318269d010. 23059497

[30] S. Abe , A. Sakai , A. Masuda , et al., “Advantage of Endoscopic Papillectomy for Ampullary Tumors as an Alternative Treatment for Pancreatoduodenectomy,” Scientific Reports 12, no. 1 (2022): 15134, 10.1038/s41598-022-19439-3. 36071180 PMC9452518

[31] K. Ito , N. Fujita , Y. Noda , et al., “Impact of Technical Modification of Endoscopic Papillectomy for Ampullary Neoplasm on the Occurrence of Complications,” Digestive Endoscopy 24, no. 1 (2012): 30–35, 10.1111/j.1443-1661.2011.01161.x. 22211409

[32] S. Irani , A. Arai , K. Ayub , et al., “Papillectomy for Ampullary Neoplasm: Results of a Single Referral Center Over a 10‐Year Period,” Gastrointestinal Endoscopy 70, no. 5 (2009): 923–932, 10.1016/j.gie.2009.04.015. 19608181

[33] M. Kobayashi , S. Ryozawa , H. Iwano , et al., “The Usefulness of Wire‐Guided Endoscopic Snare Papillectomy for Tumors of the Major Duodenal Papilla,” PLoS One 14, no. 1 (2019): e0211019, 10.1371/journal.pone.0211019. 30673748 PMC6343902

[34] Y. Takada , T. Ishikawa , K. Yamao , et al., “Endoscopic Papillectomy for Ampullary Tumors in Older Patients: Balancing Oncologic Benefit and Geriatric Risk,” Digestive Endoscopy 38, no. 8 (2026): e70250, 10.1111/den.70250. 42522924 PMC13417760

[35] S. Kiriyama , K. Kozaka , T. Takada , et al., “Tokyo Guidelines 2018: Diagnostic Criteria and Severity Grading of Acute Cholangitis (With Videos),” Journal of Hepato‐Biliary‐Pancreatic Sciences 25, no. 1 (2018): 17–30, 10.1002/jhbp.512. 29032610

[36] M. Yokoe , J. Hata , T. Takada , et al., “Tokyo Guidelines 2018: Diagnostic Criteria and Severity Grading of Acute Cholecystitis (With Videos),” Journal of Hepato‐Biliary‐Pancreatic Sciences 25, no. 1 (2018): 41–54, 10.1002/jhbp.515. 29032636

[37] K. J. Nass , L. W. Zwager , M. van der Vlugt , et al., “Novel Classification for Adverse Events in GI Endoscopy: The AGREE Classification,” Gastrointestinal Endoscopy 95, no. 6 (2022): 1078–1085.e8, 10.1016/j.gie.2021.11.038. 34890695

[38] M. Sugimoto , M. Murata , K. Shionoya , T. Tsuchiya , and T. Itoi , “Delayed Bleeding After Endoscopic Sphincterotomy in Patients Receiving Anticoagulants,” Digestive Endoscopy 37, no. 7 (2025): 733–746, 10.1111/den.15016. 40040592

[39] S. Mukai , Y. Takeyama , T. Itoi , et al., “Clinical Practice Guidelines for Post‐ERCP Pancreatitis 2023,” Digestive Endoscopy 37, no. 6 (2025): 573–587, 10.1111/den.15004. 40132896

[40] N. Nguyen , J. N. Shah , and K. F. Binmoeller , “Outcomes of Endoscopic Papillectomy in Elderly Patients With Ampullary Adenoma or Early Carcinoma,” Endoscopy 42, no. 11 (2010): 975–977, 10.1055/s-0030-1255875. 21072717

[41] Y. Takada , H. Kawashima , E. Ohno , et al., “The Impact of the Age‐Adjusted Charlson Comorbidity Index as a Prognostic Factor for Endoscopic Papillectomy in Ampullary Tumors,” Journal of Gastroenterology 57, no. 3 (2022): 199–207, 10.1007/s00535-022-01853-z. 35098349

[42] J. Kandler , T. Essing , A. Mertens , et al., “Endoscopic Papillectomy in Germany: A Comprehensive Analysis of Clinical Trends and Hospital Mortality in Germany,” Scandinavian Journal of Gastroenterology 60, no. 12 (2025): 1160–1170, 10.1080/00365521.2025.2557416. 40988622

[43] H. Kawashima , “Prevention of Delayed Bleeding in Endoscopic Papillectomy,” Digestive Endoscopy 38, no. 1 (2026): e70066, 10.1111/den.70066. 41251681

[44] L. Liu , H. Liu , and Z. Feng , “A Narrative Review of Postoperative Bleeding in Patients With Gastric Cancer Treated With Endoscopic Submucosal Dissection,” Journal of Gastrointestinal Oncology 13, no. 1 (2022): 413–425, 10.21037/jgo-21-466. 35284137 PMC8899759

[45] J. Zhao , Z. Sun , J. Liang , S. Guo , and D. Huang , “Endoscopic Submucosal Dissection for Early Gastric Cancer in Elderly vs. Non‐Elderly Patients: A Systematic Review and Meta‐Analysis,” Frontiers in Oncology 11 (2022): 718684, 10.3389/fonc.2021.718684. 35096560 PMC8792970

[46] R. Laugier , J. P. Bernard , P. Berthezene , and P. Dupuy , “Changes in Pancreatic Exocrine Secretion With Age: Pancreatic Exocrine Secretion Does Decrease in the Elderly,” Digestion 50, no. 3–4 (1991): 202–211, 10.1159/000200762. 1812045

[47] K. Li , J. Bian , Y. Xiao , et al., “Changes in Pancreatic Senescence Mediate Pancreatic Diseases,” International Journal of Molecular Sciences 24, no. 4 (2023): 3513, 10.3390/ijms24043513. 36834922 PMC9962587

[48] S. Fukuhara , M. Kato , E. Iwasaki , et al., “External Drainage of Bile and Pancreatic Juice After Endoscopic Submucosal Dissection for Duodenal Neoplasm: Feasibility Study (With Video),” Digestive Endoscopy 33, no. 6 (2021): 977–984, 10.1111/den.13907. 33258135

[49] D. Rosso , G. Carnazzo , L. Giarelli , L. Motta , and D. Maugeri , “Atherosclerosis and Pancreatic Damage,” Archives of Gerontology and Geriatrics 32, no. 2 (2001): 95–100, 10.1016/s0167-4943(00)00088-1. 11313100

[50] F. Finkelmeier , A. Tal , M. Ajouaou , et al., “ERCP in Elderly Patients: Increased Risk of Sedation Adverse Events but Low Frequency of Post‐ERCP Pancreatitis,” Gastrointestinal Endoscopy 82, no. 6 (2015): 1051–1059, 10.1016/j.gie.2015.04.032. 26089104

[51] N. Maitin‐Casalis , T. Neeman , and A. Thomson , “Protective Effect of Advanced Age on Post‐ERCP Pancreatitis and Unplanned Hospitalisation,” Internal Medicine Journal 45, no. 10 (2015): 1020–1025, 10.1111/imj.12844. 26122669

[52] P. Katsinelos , G. Lazaraki , G. Chatzimavroudis , et al., “The Impact of Age on the Incidence and Severity of Post‐Endoscopic Retrograde Cholangiopancreatography Pancreatitis,” Annals of Gastroenterology 31, no. 1 (2018): 96–101, 10.20524/aog.2018.0203. 29333073 PMC5759619

[53] S. O. Ross and C. E. Forsmark , “Pancreatic and Biliary Disorders in the Elderly,” Gastroenterology Clinics of North America 30, no. 2 (2001): 531–545, 10.1016/s0889-8553(05)70194-1. 11432304

[54] K. Takeda , M. Yokoe , T. Takada , et al., “Assessment of Severity of Acute Pancreatitis According to New Prognostic Factors and CT Grading,” Journal of Hepato‐Biliary‐Pancreatic Sciences 17, no. 1 (2010): 37–44, 10.1007/s00534-009-0213-4. 20012329

[55] S. Zhang , Z. Chen , C. Hu , et al., “The Clinical Characteristics and Outcomes of Acute Pancreatitis Are Different in Elderly Patients: A Single‐Center Study Over a 6‐Year Period,” Journal of Clinical Medicine 13, no. 16 (2024): 4829, 10.3390/jcm13164829. 39200971 PMC11355819

[56] U. Hamdani , R. Naeem , F. Haider , et al., “Risk Factors for Colonoscopic Perforation: A Population‐Based Study of 80118 Cases,” World Journal of Gastroenterology 19, no. 23 (2013): 3596–3601, 10.3748/wjg.v19.i23.3596. 23801860 PMC3691036

[57] L. W. Day , A. Kwon , J. M. Inadomi , L. C. Walter , and M. Somsouk , “Adverse Events in Older Patients Undergoing Colonoscopy: A Systematic Review and Meta‐Analysis,” Gastrointestinal Endoscopy 74, no. 4 (2011): 885–896, 10.1016/j.gie.2011.06.023. 21951478 PMC3371336

[58] V. Lohsiriwat , S. Sujarittanakarn , T. Akaraviputh , N. Lertakyamanee , D. Lohsiriwat , and U. Kachinthorn , “What Are the Risk Factors of Colonoscopic Perforation?,” BMC Gastroenterology 9 (2009): 71, 10.1186/1471-230X-9-71. 19778446 PMC2760570

[59] A. K. Bohra , L. McKie , and T. Diamond , “Transduodenal Excision of Ampullary Tumours,” Ulster Medical Journal 71, no. 2 (2002): 121–127. 12513008 PMC2475303

[60] A. Di Giorgio , S. Alfieri , F. Rotondi , et al., “Pancreatoduodenectomy for Tumors of Vater's Ampulla: Report on 94 Consecutive Patients,” World Journal of Surgery 29, no. 4 (2005): 513–518, 10.1007/s00268-004-7498-x. 15776300

[61] D. L. Cahen , P. Fockens , L. T. de Wit , G. J. Offerhaus , H. Obertop , and D. J. Gouma , “Local Resection or Pancreaticoduodenectomy for Villous Adenoma of the Ampulla of Vater Diagnosed Before Operation,” British Journal of Surgery 84, no. 7 (1997): 948–951, 10.1002/bjs.1800840711. 9240132

[62] P. H. Jordan, Jr. , G. Ayala , W. R. Rosenberg , and B. M. Kinner , “Treatment of Ampullary Villous Adenomas That May Harbor Carcinoma,” Journal of Gastrointestinal Surgery 6, no. 5 (2002): 770–775, 10.1016/s1091-255x(02)00040-9. 12399068

[63] T. C. Tran and G. C. Vitale , “Ampullary Tumors: Endoscopic Versus Operative Management,” Surgical Innovation 11, no. 4 (2004): 255–263, 10.1177/155335060401100409. 15756395

[64] M. Dubois , I. Labgaa , G. Dorta , and N. Halkic , “Endoscopic and Surgical Ampullectomy for Non‐Invasive Ampullary Tumors: Short‐Term Outcomes,” Bioscience Trends 10, no. 6 (2017): 507–511, 10.5582/bst.2016.01193. 27990004

[65] H. Kawashima , E. Ohno , T. Ishikawa , et al., “Endoscopic Papillectomy for Ampullary Adenoma and Early Adenocarcinoma: Analysis of Factors Related to Treatment Outcome and Long‐Term Prognosis,” Digestive Endoscopy 33, no. 5 (2021): 858–869, 10.1111/den.13881. 33107134

[66] H. Kawashima , “Outcomes of Endoscopic Papillectomy Should be Evaluated Not Only Based on Short‐Term Results but Also Long‐Term Prognosis,” Digestive Endoscopy 37, no. 9 (2025): 952–954, 10.1111/den.15044. 40470683

[67] A. D. Singh , A. Bhatt , A. Joseph , et al., “Natural History of Ampullary Adenomas in Familial Adenomatous Polyposis: A Long‐Term Follow‐Up Study,” Gastrointestinal Endoscopy 95, no. 3 (2022): 455–467.e3, 10.1016/j.gie.2021.09.036. 34624304

