# Supplementary material for: Endoscopic Papillectomy May Be a Feasible Treatment Option for Elderly Patients With Ampullary Tumors: A Multicenter Retrospective Study
Source: Dig Endosc. 2026 Sep 10;38(9):e70284. doi: 10.1111/den.70284 (PMC13559748; doi:10.1111/den.70284)
Supplement: Supplementary file 1 [file DEN-38-0-s001.docx]

Supplementary Table S1. Details of EP procedure

| Item | Details |
| --- | --- |
| Endoscopist | Experienced pancreatobiliary endoscopist with >500 ERCP procedures. |
| Duodenoscope | JF-260V, TJF-260V, or TJF-Q290V (Olympus, Tokyo, Japan). |
| Sedation and analgesia | Standard regimen: intravenous midazolam (2–10 mg; Astellas Pharma, Tokyo, Japan) for sedation and pentazocine (15 mg; Maruishi Pharma, Osaka, Japan) for analgesia, with diazepam (5 mg; Takeda Pharma, Tokyo, Japan) added as required.  In patients anticipated to require deeper sedation: propofol (Maruishi Pharma, Osaka, Japan) for sedation and pethidine hydrochloride (Takeda Pharmaceutical Co., Tokyo, Japan) for analgesia.  All patients were monitored continuously with blood-pressure measurement, electrocardiography, and pulse oximetry (SpO₂) throughout the procedure. Capnography (CO₂ monitoring) was introduced at Yokohama City University Hospital in 2017 and has been used routinely at that institution since then; it was not used at the other participating institutions during the study period. |
| Submucosal injection | Not performed. |
| Snare | Captivator II (Boston Scientific Corporation, Tokyo, Japan); size selected according to tumor diameter. |
| Electrosurgical unit | • Endocut mode: ICC200 or VIO300D (ERBE Elektromedizin, Tübingen, Germany); 80–120 W, Effect 3.  • Pulse-cut mode: ESG100 (Olympus, Tokyo, Japan); 80 W. |
| Prophylactic ulcer closure | Endoscopic hemoclips applied to the caudal side of the post-EP ulcer to reduce delayed bleeding risk.  • EZ clip (Olympus, Tokyo, Japan).  • SureClip (Micro-Tech Corporation, Nanjing, China). |
| Intra-procedural hemostasis | At the discretion of the endoscopist:  • Argon plasma coagulation.  • Clipping.  • Topical hemostatic agents: PuraStat (self-assembling peptide; 3D Matrix Europe SAS, Caluire-et-Cuire, France) or thrombin (Mochida Pharmaceutical Co., Tokyo, Japan). |
| Prophylactic stenting | Attempted in all patients.  • Biliary drainage: 7-Fr plastic stent.  • Pancreatic duct drainage: 5-Fr plastic stent. |
| Periprocedural medications | Acid-suppressive agents were administered to all patients during the periprocedural period.  Prophylactic nafamostat was administered immediately prior to the EP. |

EP, endoscopic papillectomy; ERCP, endoscopic retrograde cholangiopancreatography; Fr, French.

Supplementary Table S2. Definitions of EP-related complications

| Complications | Definition |
| --- | --- |
| Immediate bleeding | Bleeding requiring endoscopic hemostasis immediately after EP. |
| Delayed bleeding | Clinical evidence of melena or hematemesis requiring endoscopic intervention, or a decrease in hemoglobin of ≥2 g/dL following EP. |
| Post-EP pancreatitis (diagnosis) | New or worsening abdominal pain persisting >24 hours, together with serum amylase >3× ULN after 24 hours. |
| Post-EP pancreatitis (severity) | Post-EP pancreatitis was classified as severe when it required >10 days of treatment or was associated with necrotizing pancreatitis. |
| Duodenal perforation | Presence of free air in the peritoneal or retroperitoneal space. |
| Biliary tract infection | Cholangitis and cholecystitis were diagnosed according to the Tokyo Guidelines 2018. |

EP, endoscopic papillectomy; ULN, upper limit of normal.

Supplementary Table S3. AGREE classification for grading the severity of adverse events

| Grading | Definition |
| --- | --- |
| No adverse event | • A telephone contact with the general practitioner, outpatient clinic, or endoscopy service without any intervention, or  • Extended observation of the patient after the procedure, <3 hours, without any intervention. |
| Grade I | Adverse events with any deviation of the standard post-procedural course, without the need for pharmacologic treatment or endoscopic, radiologic, or surgical interventions.  • Presentation at the emergency ward, without any intervention, or  • Hospital admission (<24 hours), without any intervention, or  • Allowed therapeutic regimens are drugs as antiemetics, antipyretics, analgesics, and electrolytes, or  • Allowed diagnostic tests: radiology and laboratory tests. |
| Grade II | • Adverse events requiring pharmacologic treatment with drugs other than those allowed for grade I adverse events (i.e., antibiotics, antithrombotics, etc.), or  • Blood or blood product transfusions, or  • Hospital admission for more than 24 hours. |
| Grade III | Adverse events requiring endoscopic, radiologic, or surgical intervention. |
| Grade IIIa | Endoscopic or radiologic intervention. |
| Grade IIIb | Surgical intervention. |
| Grade IV | Adverse events requiring intensive care unit/critical care unit admission. |
| Grade IVa | Single-organ dysfunction (including dialysis). |
| Grade IVb | Multiorgan dysfunction. |
| Grade V | Death of the patient. |

An adverse event was defined as any negative outcome for a patient that prevented completion of the planned procedure or caused any deviation from the standard postprocedural course. Adapted from the AGREE classification by Nass et al.
